# You shall not pass! A Chromatin barrier story in plants

**DOI:** 10.3389/fpls.2022.888102

**Published:** 2022-09-20

**Authors:** Florent Velay, Louis-Valentin Méteignier, Christophe Laloi

**Affiliations:** ^1^Aix Marseille Université, CEA, CNRS, Biosciences and Biotechnologies Institute of Aix-Marseille (BIAM), Equipe de Luminy de Génétique et Biophysique des Plantes, Marseille, F-13009, France; ^2^EA2106 Biomolécules et Biotechnologies Végétales, Université de Tours, Tours, France

**Keywords:** insulators, topoisomerase, barrier function, heterochromatin spreading, chromatin domains, chromatin islands

## Abstract

As in other eukaryotes, the plant genome is functionally organized in two mutually exclusive chromatin fractions, a gene-rich and transcriptionally active euchromatin, and a gene-poor, repeat-rich, and transcriptionally silent heterochromatin. In *Drosophila* and humans, the molecular mechanisms by which euchromatin is preserved from heterochromatin spreading have been extensively studied, leading to the identification of insulator DNA elements and associated chromatin factors (insulator proteins), which form boundaries between chromatin domains with antagonistic features. In contrast, the identity of factors assuring such a barrier function remains largely elusive in plants. Nevertheless, several genomic elements and associated protein factors have recently been shown to regulate the spreading of chromatin marks across their natural boundaries in plants. In this minireview, we focus on recent findings that describe the spreading of chromatin and propose avenues to improve the understanding of how plant chromatin architecture and transitions between different chromatin domains are defined.

## Introduction

It has been almost a century since the crucial role of chromatin functional compartmentation in gene expression was revealed by the study of the *white* mutation in *Drosophila* that causes white rather than red eye pigmentation. By using X-ray mutagenesis, Muller identified partial suppressors of *white* in which some facets of the eye remained white while others developed the red pigmentation, suggesting that the *white* allele itself was not mutated (Muller, 1930). It was much later shown that X-rays induced chromosomal rearrangements that resulted in a change of position of *white* from its original euchromatic position to the centromeric heterochromatin (Zhimulev et al., 1986, 1988; Umbetova et al., 1991). Heterochromatin is characterized by its high compactness and repressive epigenetic marks, which include DNA methylation and specific post-translation modifications of histones (or histone marks). DNA sequences in heterochromatin include many transposable elements (TEs) whose replication and reactivation are therefore prevented. Since Muller’s pioneering work, a series of studies have revealed that, besides the particular context of X-ray-induced chromosomal rearrangements, heterochromatin can spread stochastically into the adjacent euchromatin and effectively repress the expression of underlying genes. Some DNA sequences and associated protein modules that normally prevent heterochromatin spreading and maintain transitions between distinct chromatin domains have been identified and named “barrier insulators.” Insulator functions, however, encompass a wider range of mechanisms than the simple delimitation of antagonistic chromatin domains. Indeed, the term “insulator” is likewise attributed to factors that regulate the accessibility of enhancers and silencers to promoters or to factors participating in the structuration of DNA loops such as Topologically-Associated Domains (TAD; Ali et al., 2016). In this minireview, we focus on barrier insulators. We briefly introduce the establishment and maintenance of heterochromatin in plants (for a more complete review, see Law and Jacobsen, 2010), and subsequently present several loci within which a barrier activity is carried out in plants. Finally, we discuss several strategies aimed at increasing our knowledge of this understudied function in plants.

## What can we learn from animal and yeast models about chromatin barrier formation in plants?

In animals and yeasts, the barrier function preventing the spreading of chromatin features beyond their natural boundaries relies on the presence of DNA sequences and their related DNA binding proteins such as the Transcription Factors for polymerase III B and C (TFIIIB, TFIIIC), which limit the spreading of the Silent Information Regulatory proteins (Sir) promoting the establishment of silenced chromatin in *Saccharomyces cerevisiae* (Sun et al., 2011). Similarly, the Upstream Stimulatory Factors 1 and 2 (USF1, USF2) bind the 5′HS4 insulator element and are necessary to avoid the spreading of neighboring heterochromatin in chicken erythrocytes (Ghirlando et al., 2012). Another example is the Heterochromatin Protein 1 (HP1), which binds H3K9me, a typical heterochromatin histone post-translational modification, and is required to prevent the spreading of H3K9me and H3K27me3 across natural boundaries in fungi (Stunnenberg et al., 2015; Jamieson et al., 2016). However, the unique homolog of HP1 in Arabidopsis, LHP1, regulates the deposition of H3K27me3 but does not prevent its spreading (Veluchamy et al., 2016). In general, no homologs of barrier elements described in animals and yeast have been reported to have similar functions in plants. Despite the absence of such barrier insulators in the literature, plant genomes have well-delineated euchromatin/heterochromatin compartmentation (Schoborg and Labrador, 2010). This raises the questions of the existence and identity of plant-specific barrier insulators.

## Heterochromatin establishment and maintenance in plants

In plants, the histone modification H3K9me2 is a hallmark of heterochromatin. It is established and maintained by SUPPRESSORS OF VARIEGATION HOMOLOG (SUVH) histone methyltransferases (Ebbs et al., 2005). CHH and CHG (where H is any base except G) DNA methylation and H3K9me2 establishment were shown to be maintained through a positive feedback loop: SUVH proteins bind preferentially methylated non-CG cytosines and establish H3K9me2. Reciprocally, plant-specific CHROMOMETHYLASES 2 and 3 (CMT2/3) bind H3K9me2 and establish DNA methylation on CHH (CMT2) and CHG (CMT3) (Papa, 2001; Jackson et al., 2002, 2004; Johnson et al., 2007, 2014; Du et al., 2012; Stroud et al., 2014; Li et al., 2018b). Maintenance of CG methylation is ensured by the METHYLTRANSFERASE 1 (MET1; Cokus et al., 2008). *De novo* methylation of DNA (CG, CHG, and CHH) involves the RNA-directed DNA methylation pathway (RdDM) as well as the DOMAINS REARRANGED METHYLASE 1 and 2 (DRM1/2) proteins (Cao and Jacobsen, 2002a,b).

The initiation of the silencing of an active TE is mediated by non-canonical RdDM mechanisms based on the activity of RNA polymerase II (Nuthikattu et al., 2013): the RNA DEPENDENT RNA POLYMERASE 6 (RDR6) and the DICER LIKE 3 (DCL3) pathways. In *Arabidopsis thaliana*, the RDR6 pathway starts with the processing of TE mRNAs by RDR6 in order to produce a double-stranded RNA, which is cleaved by the endonucleases DCL2 and DCL4 (McCue et al., 2012). A pool of 21–22 nt siRNAs is thus generated and bound by the ARGONAUTE 1 (AGO1) protein (McCue et al., 2012), creating a complex able to bind the long non-coding RNAs (lncRNAs) produced *in situ* by Pol V. This complex recruits the DOMAINS REARRANGED METHYLTRANSFERASE 2 (DRM2), which catalyzes *de novo* cytosine methylation (Cao and Jacobsen, 2002b; Zhong et al., 2014). In the DCL3 pathway, the production of 24-nt siRNAs is independent of RDR2 and RDR6 and generated directly by DCL3, probably thanks to internal hairpin structures (Panda et al., 2016). Maintenance of TE silencing first depends on the addressing of Pol IV at H3K9me2 and unmethylated H3K4 sites. Following recruitment, Pol IV, with the help of RDR2, produces a pool of double-stranded precursor RNAs, which are then cleaved by DCL3 to generate 24 nt siRNAs (Law et al., 2011, 2013; Zhang et al., 2013; Li et al., 2015b; Singh et al., 2019). In the second phase, Pol V produces a second pool of lncRNAs which can associate with AGO4 or AGO6 loaded with the RNAs produced by Pol IV, thus allowing the recruitment of DRM2 (Wierzbicki et al., 2009; Matzke and Mosher, 2014; Duan et al., 2015). This model was recently refined after the demonstration that Pol IV transcripts can also guide the DNA methylation process in a DICER-independent manner at several loci (Yang et al., 2016; Ye et al., 2016; Kuo et al., 2017).

## Heterochromatin spreading is counteracted by active DNA demethylation in *Arabidopsis thaliana*

**Figure 1:**
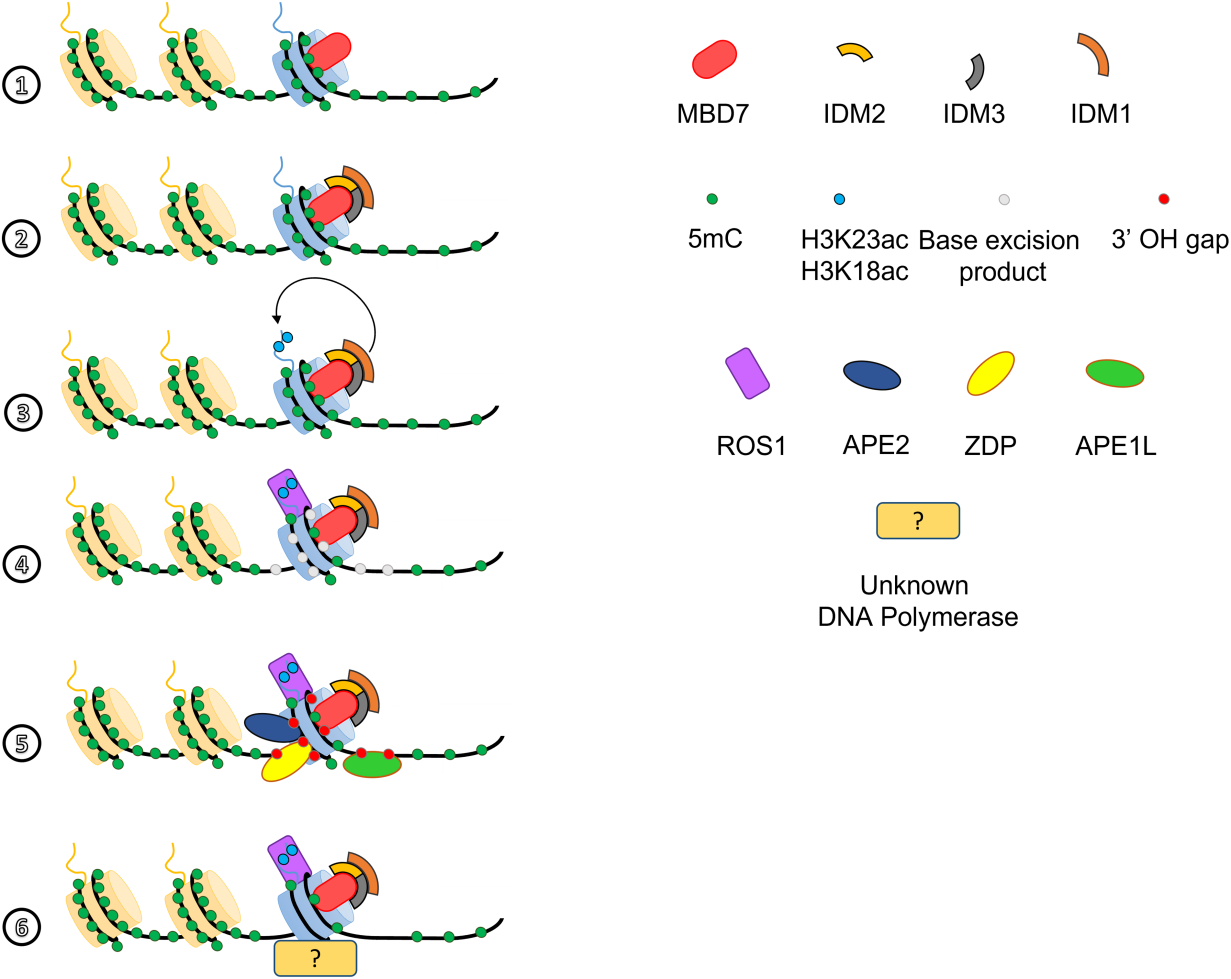
Methyl-CpG-Binding Domain 7 (MBD7), ROS1, and the base excision repair pathway act together to locally demethylate DNA. MBD7 binds highly methylated loci (1) and, through binding of IDM2 and IDM3, targets the histone methyl transferase IDM1 to mCG-dense regions (2). IDM1 ensures *in situ* deposition of H3K18ac and H3K23ac marks (3), which allows ROS1 to be addressed to hypermethylated regions (4; Lang et al., 2015). After removal of 5mC by ROS1 (4), the excision product can be processed by APE1L, APE2, and ZDP to create a 5’P-3’OH gap (5), which is then filled by a yet unknown DNA polymerase and sealed by DNA ligase I (6; Li et al., 2015c,d, 2018a).

Several studies have shown that plants are prone to heterochromatin spreading (Saze et al., 2008; Miura et al., 2009; Eichten et al., 2012; Lang et al., 2015; Noshay et al., 2019). However, there is still little evidence for the mechanisms involved in spreading heterochromatin into neighboring regions or, conversely, in preventing heterochromatin spreading by maintaining boundaries between different chromatin domains with antagonistic features. In Arabidopsis, mutations in RdDM factors suppressed the epidermal patterning defects observed in a mutant of the DNA glycosylase/lyase ROS1, a phenotype caused by the hypermethylation of the *EPIDERMAL PATTERNING FACTOR 2* gene (Yamamuro et al., 2014). Another DNA glycosylase, DEMETER (DME), is required for the expression of imprinted genes localized near TEs, in the endosperm of Arabidopsis (Gehring et al., 2009). Mutations in DEMETER-Like (DML) 2, 3 and ROS1 are associated with increased DNA methylation of promoter and terminator regions (Lister et al., 2008). In addition, mutations in *CMT3* and *SUVHs* suppressed the phenotypes resulting from the mutation of the gene encoding the H3K9 demethylase INCREASE IN BONZAI METHYLATION 1 (IBM1; Saze et al., 2008). RdDM and SUVH/CMT activities therefore appear to be counteracted by several DNA or histone demethylases as ROS1, DME, DMLs, and IBM1, but how these different antagonistic actions are orchestrated genome-wide is poorly understood. Nevertheless, a recent genetic screen revealed that mutants of Methyl-CpG-Binding Domain 7 (MBD7) and Increased DNA Methylation 3 (IDM3) fail to express a transgene composed of the sucrose transporter SUC2 coding sequence under the control of the 35S promoter. Subsequently, bisulfite sequencing analysis revealed that in a WT plant, 35S promoter accumulates DNA methylations in the region flanking the coding sequence of SUC2, whereas in *mbd7* and *idm3* mutants, the DNA methylations spread toward the 5′ region of the 35S promoter (Lang et al., 2015). In the model established by Lang and colleagues, MBD7 binds to mCG dense regions and interacts with IDM2 and IDM3. The complex composed of MBD7-IDM2-IDM3 would then recruit the histone acetyltransferase IDM1 to highly methylated loci, leading to *in situ* deposition of H3K23ac and H3K18ac marks, thus creating a permissive chromatin environment for ROS1 to locally remove the methylated cytosines in the regions bound by MBD7 (Lang et al., 2015; Figure 1). Subsequently, the base excision repair pathway, composed of the Apurinic/Apyrimidinic Endonucleases (APE1L and APE2) and the Zinc Finger DNA 3’ Phosphoesterase (ZDP), processes the excision product of the 5 mC and generates a 3’OH gap which is filled by an unknown DNA polymerase (Li et al., 2015c, 2018a; Figure 1). Thus, the combined action of these different actors is thought to prevent the diffusion of DNA methylations beyond their natural boundaries in plants. Very recently, the same genetic screen that allowed the identification of MBD7 has led to the discovery of another barrier factor, AGDP3. AGDP3 associates with H3K9me2 in a pH-dependent manner and protects several loci against ectopic DNA methylation. Since the targeting of ROS1 and subsequent demethylation of targeted loci appear to be dependent on AGDP3, it reinforces the idea that active demethylation acts downstream of the barrier activity (Zhou et al., 2021).

## Euchromatin islands

**Figure 2:**
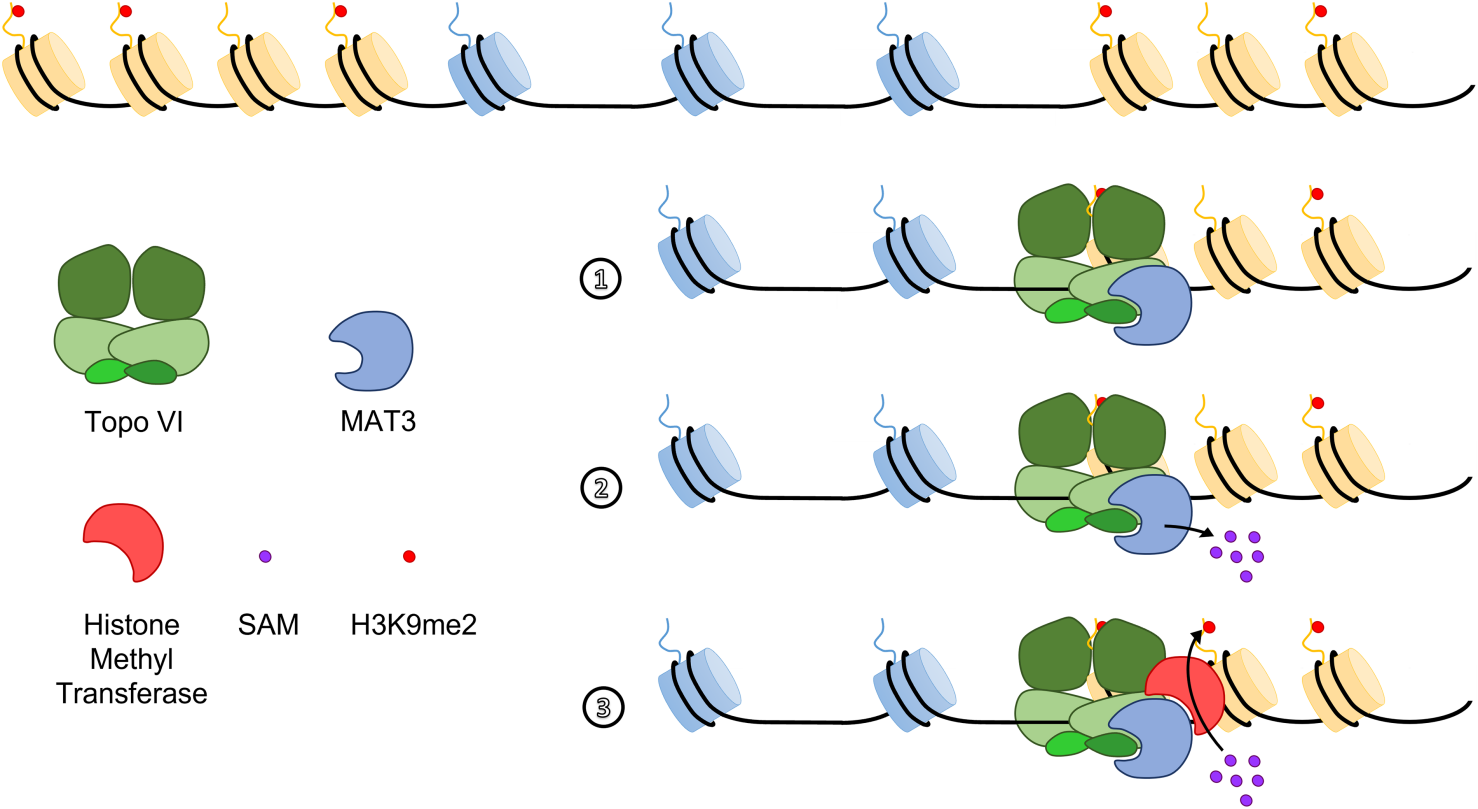
A hypothetical model for topoisomerase VI (Topo VI)-dependent insulation at Euchromatin Islands. The BIN4 subunit of Topo VI interacts with METHIONINE ADENOSYL TRANSFERASE 3 (MAT3) and is required to exclude MAT proteins from EIs (1). MAT3 localization at heterochromatin edges would allow local production of *S-*Adenosyl Methionine [SAM, (2)], the main methyl donor in methylation reactions, which is processed by a yet unknown histone methyltransferase to ensure *in situ* deposition of the repressive mark H3K9me2 in heterochromatin regions (3; Méteignier et al., 2022).

Euchromatin islands (EIs) were initially defined in humans as small euchromatin regions within large heterochromatin regions, which contain expressed genes, are depleted for H3K9me2, and are enriched for CTCF binding sites (Wen et al., 2012). Despite the absence of CTCF homologs, similar EI structures were described in Arabidopsis (Méteignier et al., 2022), rice (Wu et al., 2011), and barley (Baker et al., 2015) and represent ideal context to study heterochromatin spreading and identify barrier insulator elements. No consensus DNA sequences at the boundaries between EIs and the flanking heterochromatin regions, which could serve as barrier insulator sequences, were reported. However, chromatin state 8, an AT-rich heterochromatic state, less inaccessible and dense than the classic GC-rich heterochromatic state 9 (Sequeira-Mendes et al., 2014), was always present in the proximal borders of Arabidopsis EIs (Méteignier et al., 2022). In Arabidopsis, mutants of the topoisomerase VI (Topo VI) complex failed to express EI genes at their physiological levels. Strikingly, EIs are invaded by H3K9me2 in a hypomorphic Topo VI mutant, which suggests that Topo VI participates in a chromatin barrier function in Arabidopsis (Méteignier et al., 2022). Interestingly, the BRASSINOSTEROID INSENSITIVE 4 (BIN4) subunit of Topo VI directly interacts with METHIONINE ADENOSYL TRANSFERASE 3 (MAT3), the major methyl donor of methylation reactions (Meng et al., 2018; Méteignier et al., 2022). In addition, MAT3 was required for H3K9me2 deposition at TEs, and Topo VI was required to exclude MAT3 from EIs, thereby providing a possible mechanistic explanation to the participation of Topo VI in a barrier function (Figure 2). The spreading of H3K9me2 over EIs was, however, not accompanied by an increase in cytosine methylation levels in CHG or CHH contexts, suggesting that Topo VI is involved in controlling the spreading of some chromatin marks rather than heterochromatin itself (Méteignier et al., 2022). This uncoupling between H3K9me2 and DNA methylation has been observed in several other specific contexts. For instance, in triploid Arabidopsis seeds, small euchromatic AT-rich TEs overaccumulate H3K9me2 but lose CHH methylation (no change in CHG nor CG), which correlates with altered expression of neighboring genes (Jiang et al., 2017). This result suggests that H3K9me2 may repress RdDM, as it was observed in another independent study (Zemach et al., 2013). Another study in *Eutrema salsugineum*, which lacks CMT3 and gene body cytosine methylation, has highlighted a similar disruption of the reinforcement loops between H3K9me2 and non-CG methylation. In this organism, the ectopic expression of AtCMT3 increased CHG methylation over repetitive sequences containing pre-existing H3K9me2. Additionally, ectopic gene body CHG methylations at loci devoid of pre-existing H3K9me2 were also reported. In the latter case, the CHG methylations were stably inherited through generations, but did not lead to subsequent induction of H3K9me2 deposition (Wendte et al., 2019). Hence, in the context of Topo VI-dependent barrier, H3K9me2 might also be established and maintained in a CHG-independent manner by unknown histone methyl transferases.

## mCHH islands

In the heterochromatin-rich maize genome, gene-flanking regions are heavily methylated in CHH context and were therefore called mCHH islands (Gent et al., 2013). Mutation in the *RDR2* ortholog *MOP1* decreased the production of 24-nt siRNAs by 100-fold and altered the formation of mCHH islands (Nobuta et al., 2008; Gent et al., 2014), revealing the important role of RdDM for CHH methylation in maize, which lacks known orthologs of *CMT2* (Bewick et al., 2017). Epigenome mapping of maize revealed that mCHH islands are specific to transition zones between CG/CHG/H3K9me2-rich intergenic regions and H3K4me3-rich gene extremities. In addition, the same study suggested increased chromatin accessibility around promoters and terminators of genes adjacent to mCHH islands (Li et al., 2015a). These studies thus identified mCHH islands as good candidates for barrier insulator elements. Interestingly, methylome analyses of *mop1* and *mop3* mutants revealed that loss of mCHH islands was not associated with altered expression of neighboring genes, but rather with decreased CG/CHG methylation and elevated expression of adjacent TEs. This suggests that mCHH islands are essential for preserving silent TEs from activation by euchromatin spreading (Li et al., 2015a). Reinforcing this idea, a recent study reports that disruption of *mop1* leads to loss of DNA methylation at TEs which are then susceptible to reactivation upon heat stress (Guo et al., 2021). Since then, mCHH islands have been identified in the genomes of many angiosperms (Zemach et al., 2010; Niederhuth et al., 2016; Corem et al., 2018; Martin et al., 2021). In Poaceae, mCHH islands are enriched at the extremities of 39% of genes, particularly at their 5′ extremity in smaller genomes. Among gene expression, gene length, distance from a TE, and gene body methylation, TE proximity and absence of gene body methylation were the strongest predictors for the presence of 5′ CHH islands (Martin et al., 2021). In Arabidopsis, the edges of long heterochromatic TEs are hypermethylated in CHH context and siRNAs overaccumulate at these positions (Zemach et al., 2013). Thus, mCHH islands could exert a similar function in very different genomes by establishing nearby TEs. Methylation in CHH context increases locally in the proximity of genes and overlaps almost exclusively with neighboring TEs in response to phosphate starvation in rice. Moreover, the large majority of genes containing differentially methylated regions after a long-term phosphate starvation are upregulated (Secco et al., 2015). A similar result has recently been observed in tomato (Tian et al., 2021). Such a dynamic appearance of mCHH islands in the vicinity of highly expressed genes neighboring TEs is consistent with the hypothesis that mCHH islands prevent euchromatin spreading to adjacent TEs. Thus, mCHH islands might constitute a conserved epigenetic response aimed at protecting plant genomes against opportunistic reactivation of TEs during stress exposure. However, to assert that mCHH islands constitute a genuine and widespread barrier against euchromatin spreading, further studies are required, such as the determination of H3K4me3 distribution in the neighborhood of mCHH islands in *mop* mutants. In addition to their likely barrier function, mCHH islands also promote the expression of genes in the vicinity to TEs through the sequential recruitment of SUVH and DNAJ domain-containing proteins (Harris et al., 2018), raising the question of the causality relationship between mCHH islands and gene expression.

## Are there common features in DNA loop formation and chromatin barriers in plants?

The interplay between the different insulator functions, such as enhancer-blocking, TAD structuration, and chromatin barrier, is rarely discussed in plants. In animals, repetitive sequences, such as tRNA genes, MIR, and Gyspy TEs, are found close to TADs and provide insulator sequences (She et al., 2010; Raab et al., 2012; Van Bortle and Corces, 2012; Wang et al., 2015). In contrast, plant TAD borders are enriched for specific transcription factor-binding sites. For instance in rice and *Marchantia polymorpha*, borders of a type of TADs are enriched for a TCP (Teosinte Branched1/Cycloidea/Proliferating cell factor) binding motif (Liu et al., 2017; Karaaslan et al., 2020). However, TCP is not required for TAD formation in Marchantia (Karaaslan et al., 2020), suggesting that TAD formation is complex and redundantly controlled in plants. Indeed, in maize, single-cell chromatin accessibility mapping has revealed that TCP-binding sites are enriched in co-accessible *cis*-regulatory regions, which corresponded to the borders of chromatin loops. Other transcription factor-binding sites were found at a similar location, such as APETALA2/ETHYLENE-RESPONSIVE ELEMENT BINDING PROTEINS and LATERAL ORGAN BOUNDARIES DOMAIN, all representing GC-rich motifs (Marand et al., 2021). Recently, the OSH1 transcription factor, which binds the RS2-9 DNA element in rice, was proposed to act as an insulator module (Liu et al., 2022). Although this module was able to block an enhancer-promoter interaction, histone marks and TAD changes were not investigated in an *osh1* mutant. In euchromatin and at a more local scale, the BORDER plant-specific proteins are enriched at gene transcription start and end sites and are required to prevent transcriptional interference between neighboring genes (Yu et al., 2019). Taken together, these recent findings suggest potential candidates for a plant insulator function, which remains to be extensively characterized by histone modification analysis and chromatin contact mapping in mutant plants.

## Discussion

The mechanisms initiating the diffusion of heterochromatin in plants are currently unclear. Heterochromatin spreading observed in *ros1* is highly dependent on RdDM (He et al., 2009; Yamamuro et al., 2014). However, in the context of EIs, SUVH proteins can also play a minor role in the diffusion of chromatin marks (Méteignier et al., 2022). In maize, where the vicinity of several TEs is subjected to the diffusion of heterochromatin marks (Noshay et al., 2019), neither the mutation of MOP1, a component of RdDM, nor the mutation of MET2, the chromomethylase involved in mCHG maintenance, seem to alter methylation levels in the neighborhood of these TEs (Eichten et al., 2012). Thus, there is currently no consensus on the molecular bases underlying heterochromatin spreading in plants.

The apparent absence of homologs of well-known animal insulators in plants highlighted those plants seem to use specific protein-DNA modules as well as lineage-specific proteins to ensure insulator function. Studying the barrier function in different plant models with a wide range of genome size and structure could allow a better understanding of insulation and insulator dynamics. For instance, the Topo VI-dependent barrier function at the edges of euchromatin islands has only been highlighted in Arabidopsis (Méteignier et al., 2022) and would benefit from further study in other organisms. Since Topo VI is encoded in all known plant genomes, designing a Topo VI hypomorphic or conditional mutant in maize, for instance, and studying the genome-wide distribution of H3K9me2 and other chromatin marks could provide a better insight into how genes insulate away from the adjacent TE-rich heterochromatin. Additionally, the proposed model of barrier mechanism mediated by Topo VI is incomplete. First, the physical position of Topo VI in the context of EIs and whether Topo VI binds specific DNA sequences remain elusive. However, it should be noted that in animals, topoisomerases are prone to recognize and bind particular DNA structures rather than defined DNA sequences (René et al., 2007). Moreover, the human Top IIα is addressed to the centromeres by interacting with the phosphorylated form of histone H2A at Thr 120 during mitosis (Zhang et al., 2020). These observations raise the possibility that Topo VI barrier function might also be independent of a specific nucleotide sequence. Furthermore, the putative histone methyl transferase involved in the Topo VI-controlled H3K9me2 deposition is unknown. The accumulation of RNA-seq and ChIP-seq data could make it possible to consider a data-mining approach to identify the missing factors whose mutation would also result, like Topo VI, in the repression of EI genes and changes in chromatin mark levels in EIs.

Like the barrier model involving Topo VI, the active demethylation pathway is still incomplete. Indeed, the DNA polymerase that fills the 5’P-3’OH gap resulting from the activity of the base excision repair mechanism with an unmethylated cytosine remains unknown. The discovery of the yet unidentified factors participating to the active demethylation pathway, or the Topo VI-mediated chromatin barrier, could be achieved by proximity labeling strategies allowing the identification of very transient interactions (Arora et al., 2020). Moreover, the power of the CRISPR-CAS9-based gene editing could allow inserting reporter genes within EIs, then use them in genetic screens aimed at identifying mutant lines defective in EI gene expression and thus in putative new players involved in heterochromatin containment.

## Author contributions

All authors listed have made a substantial, direct, and intellectual contribution to the work and approved it for publication.

## Funding

Work by CL was supported by the French National Research Agency (ANR-14-CE02-0010). FV is a recipient of a PhD fellowship from the French Ministry of Higher Education, Research and Innovation. Work by LVM was supported by the French National Research Agency (ANR-20-CE43-0010).

## Conflict of interest

The authors declare that the research was conducted in the absence of any commercial or financial relationships that could be construed as a potential conflict of interest.

## Publisher’s note

All claims expressed in this article are solely those of the authors and do not necessarily represent those of their affiliated organizations, or those of the publisher, the editors and the reviewers. Any product that may be evaluated in this article, or claim that may be made by its manufacturer, is not guaranteed or endorsed by the publisher.
